# 2178 Drug development core facilitates institutional collaboration and translational science innovation

**DOI:** 10.1017/cts.2018.68

**Published:** 2018-11-21

**Authors:** Gene Morse, Igor Puzanov, Andrei Gudkov, Robin DiFrancesco, William Jusko, Marc Ernstoff, James Mohler, Timothy Murphy, Robert Bies

**Affiliations:** 1 University at Buffalo, State University of New York; 2 Roswell Park Cancer Institute

## Abstract

OBJECTIVES/SPECIFIC AIMS: Drug development is a common research pursuit for basic and clinical scientists that interfaces diagnostic/therapeutic challenges with funding agencies, pharmaceutical industry, regulatory systems, and education. The University at Buffalo Clinical and Translational Science Institute (CTSI) has implemented a Drug Development Core (DDC) with goals that foster team science and collaboration, optimize laboratory use, and networks investigators. Our goals are to foster collaborations within the region and with other CTSAs. METHODS/STUDY POPULATION: The DDC met with 300 potential investigators from 14 departments and several local companies. There were 35 portal requests from 15 departments and 7 companies; 8 were from training programs. For 28 requests, a reviewer provided consultation, while 7 required discussions and review of data. DDC assisted with 15 grant applications (outcomes pending), 10 industry-related new drug development requests and 1 regulatory review. Curriculum reviews noted overlap and gaps. Cross-institute opportunities for M.D.-Ph.D. research mentoring were identified. RESULTS/ANTICIPATED RESULTS: The DDC met with 300 potential investigators from 14 departments and several local companies. There were 35 portal requests from 15 departments and 7 companies; 8 were from training programs. For 28 requests, a reviewer provided consultation, while 7 required discussions and review of data. DDC assisted with 15 grant applications (outcomes pending), 10 industry-related new drug development requests and 1 regulatory review. Curriculum reviews noted overlap and gaps. Cross-institute opportunities for M.D.-Ph.D. research mentoring were identified. DISCUSSION/SIGNIFICANCE OF IMPACT: The CTSI DDC was well received by investigators. The request process fosters collaboration among researchers with similar interests and identifies core laboratory resources that add innovation to ongoing research, funding applications, education, and interinstitutional planning.

